# The safety and efficiency of intravenous administration of tranexamic acid in coronary artery bypass grafting (CABG): a meta-analysis of 28 randomized controlled trials

**DOI:** 10.1186/s12871-019-0761-3

**Published:** 2019-06-14

**Authors:** Yanting Zhang, Yun Bai, Minmin Chen, Youfa Zhou, Xin Yu, Haiyan Zhou, Gang Chen

**Affiliations:** 1 Department of Anesthesiology, Sir Run Run Shaw Hospital, School of Medicine, Zhejiang University, Hangzhou, 310020 China; 2 Department of Anesthesiology, The Fifth People’s Hospital of Yuhang District, Hangzhou, 311100 China; 3 Department of Anesthesiology, Hangzhou Women’s Hospital, Hangzhou, 310008 China

**Keywords:** Coronary artery bypass, Postoperative complications, Tranexamic acid

## Abstract

**Background:**

The safety and efficiency of intravenous administration of tranexamic acid (TXA) in coronary artery bypass grafting (CABG) remains unconfirmed. Therefore, we conducted a meta-analysis on this topic.

**Methods:**

We searched the Cochrane Central Register of Controlled Trials (CENTRAL), PUBMED and EMBASE for randomized controlled trials on the topic. The results of this work are synthetized and reported in accordance with the PRISMA statement.

**Results:**

Twenty-eight studies met our inclusion criteria. TXA reduced the incidence of postoperative reoperation of bleeding (relative risk [RR], 0.46; 95% confidence interval [CI]; 0.31–0.68), the frequency of any allogeneic transfusion (RR, 0.64; 95% CI, 0.52–0.78) and the postoperative chest tube drainage in the first 24 h by 206 ml (95% CI − 248.23 to − 164.15). TXA did not significantly affect the incidence of postoperative cerebrovascular accident (RR, 0.93; 95%CI, 0.62–1.39), mortality (RR, 0.82; 95%CI, 0.53–1.28), myocardial infarction (RR, 0.90; 95%CI, 0.78–1.05), acute renal insufficiency (RR, 1.01; 95%CI, 0.77–1.32). However, it may increase the incidence of postoperative seizures (RR, 6.67; 95%CI, 1.77–25.20). Moreover, the subgroup analyses in on-pump and off-pump CABG, the sensitivity analyses in trials randomized more than 99 participants and sensitivity analyses that excluded the study with the largest number of participants further strengthened the above results.

**Conclusions:**

TXA is effective to reduce reoperation for bleeding, blood loss and the need for allogeneic blood products in patients undergoing CABG without increasing prothrombotic complication. However, it may increase the risk of postoperative seizures.

**Electronic supplementary material:**

The online version of this article (10.1186/s12871-019-0761-3) contains supplementary material, which is available to authorized users.

## Background

Excessive bleeding is a common complication which may lead to exposure to the risk of homologous blood transfusion and increased morbidity in patients undergoing cardiac operations [1]. Tranexamic acid (TXA), an antifibrinolytic agent, has been widely used and proved to be effective in reducing risk of blood loss and transfusion among patients undergoing cardiac surgery [2]. However, whether it reduced the incidence of reoperation for life-threatening bleeding which are strongly associated with poor outcomes after cardiac surgery remains controversial.

Despite of the effectiveness in reducing the risk of blood loss and transfusion, it may potentially increase the risk of myocardial infarction, stroke, and other thrombotic complications after cardiac surgery especially in patients undergoing coronary artery bypass grafting (CABG) surgery who are commonly characterized by systemic arteriosclerosis or stenosis [3, 4]. It was reported that TXA was associated with the increased risk of postoperative neurologic events such as stroke and seizures in cardiac surgery [5, 6]. Some studies have suggested that TXA is associated with reduction in cerebral blood flow and increase the risk of cerebral infarction [5, 7]. A multi-center study suggested that TXA was associated with a higher risk of postoperative seizures in GABG surgery [8]. A meta-analysis in 2011 has shown that TXA is associated with reduced blood transfusion in off-pump CABG surgery [9]. However, the safety of TXA in off-pump CABG surgery could not be confirmed due to the small population sample size.

An increasing number of studies that investigated the effectiveness and safety of TXA in CABG surgery have been conducted in recent years with varying results [8, 10–18]. Therefore, we conducted a meta-analysis of existing studies to estimate the safety and efficiency of TXA in CABG surgery focusing on the incidence of postoperative cerebrovascular accident, seizures and reoperation for bleeding.

## Methods

The meta-analysis was performed according to the Preferred Reporting Items for Systematic Reviews and Meta-Analyses (PRISMA) statement in this study [19].

### Search strategy

A systematic and comprehensive search was conducted in the Cochrane Central Register of Controlled Trials (CENTRAL), PUBMED and EMBASE from database established to February 8, 2018 with no language limitation. The search strategy included the following MEDLINE subject heading terms: tranexamic acid and cardiac surgical procedures. The above subject heading terms were connected by “AND”. The initial searches of PUBMED and EMBASE were unrestricted to maximize sensitivity and a filter which primarily identifies randomized controlled trials was adopted to improve the specificity. Moreover, we also checked the reference lists of relevant articles for potential relevant studies.

### Eligibility criteria

Randomized controlled trials that compared the effectiveness or safety of the intravenous administration of TXA with that of placebo in adult CABG surgery were included in this meta-analysis. Studies were eligible for inclusion, regardless of the publication language. We excluded studies which were conducted on underage patients or in which TXA was topically applied in mediastinum.

### Selection of included studies

Retrieved studies were imported into Endnote (version X7; Thomson Reuters), where duplications were detected and deleted automatically. Two authors independently scanned the titles and abstract of retrieved studies according to the established eligibility criteria to exclude the obvious unrelated studies. The full-text was further evaluated if the judgement could not easily be decided based on its title or abstract. The disagreements between reviewers were settled by a third reviewer. The relevant data of included studies was extracted by these reviewers independently using a standard data sheet. Study characteristics included author, publication year, sample size, sex ratio, type of CABG, duration of anticoagulant medication discontinued before surgery, outcome data, drug dose and treatment regimens.

### Assessment of risk of bias in included studies

The Cochrane risk of bias tool which is recommended by the Cochrane Collaboration for risk of bias assessment was adopted in this study [20]. There are seven domains in the Cochrane risk of bias tool, including the random sequence generation, allocation concealment, blinding of participants and personnel, blinding of outcome assessment, incomplete outcome data, selective reporting and other bias. The judgment of each domain is presented as “low risk”, “high risk” or “unclear risk” based on the instruction of Cochrane Collaboration. Two reviewers independently assessed each domain of included studies and any disagreements were adjudicated by a third reviewer.

### Quality of the evidence

GRADE (Grades of Recommendation, Assessment, Development and Evaluation) Working Group system was adopted to evaluate the quality of the evidence [21]. Two reviewers independently assessed the quality of each outcome. The five categories used for the GRADE quality assessment were: limitations of design, inconsistency, indirectness, imprecision, and publication bias. We used GRADE profiler (GRADEpro) software to create the “Summary of findings” table, which includes the following outcomes: incidence of postoperative cerebrovascular accident, seizures, reoperation for bleeding, mortality, myocardial infarction, acute renal insufficiency, the frequency of any allogeneic transfusions and 24-h postoperative chest tube drainage.

### Study outcomes

All outcomes were described a priori, according to the principles of the PRISMA statement. The primary outcome was incidence of postoperative cerebrovascular accident, seizures and reoperation for bleeding. The second outcomes included postoperative mortality, myocardial infarction, acute renal insufficiency, the frequency of any allogeneic transfusions and 24-h postoperative chest tube drainage.

### Statistical methods

In some studies, continuous variables was presented as median, range and/or interquartile range. To facilitate meta-analysis, we estimated the sample mean and standard deviation from median, range and/or interquartile range by using the calculator with a compiled formula recommended by Luo and colleagues [22]. The risk ratio (RR) with the corresponding 95% confidence interval (95% CI) was calculated for dichotomous data and continuous data were analyzed by using mean difference (MD) with the corresponding 95% CI. Data analyses followed the guidelines established by the Cochrane Collaboration regarding statistical methods. The statistical heterogeneity was evaluated by reviewing the *I*^*2*^ statistic and Chi^2^ test. If either the Chi^2^ test resulted in *P* < 0.10 or the *I*^*2*^ statistic was greater 50%, random-effect model was used to evaluate outcomes, otherwise a fixed-effect model was used. For all tests, two-tailed *P*-values < 0.05 were considered significant. Funnel plots were conducted to evaluate reports for publication bias when more than 10 studies were included. Considering the activation effect of cardiopulmonary bypass (CPB) on the fibrinolytic pathway, subgroup analysis was performed based on CABG with/without CPB. Moreover, Sensitivity analyses was performed in studies randomized more than 99 patients to avoid the possibility that the rare incidences of complication were underestimated due to the included studies with small population size. Sensitivity analyses that excluded the study with the largest number of participants were conducted to estimate the effect of that study on the overall effect of meta-analysis. All data analysis was conducted using Review Manager (RevMan; version 5.2), Copenhagen: The Nordic Cochrane Centre, The Cochrane Collaboration, 2012.

## Results

### Results of search

**Fig. 1:**
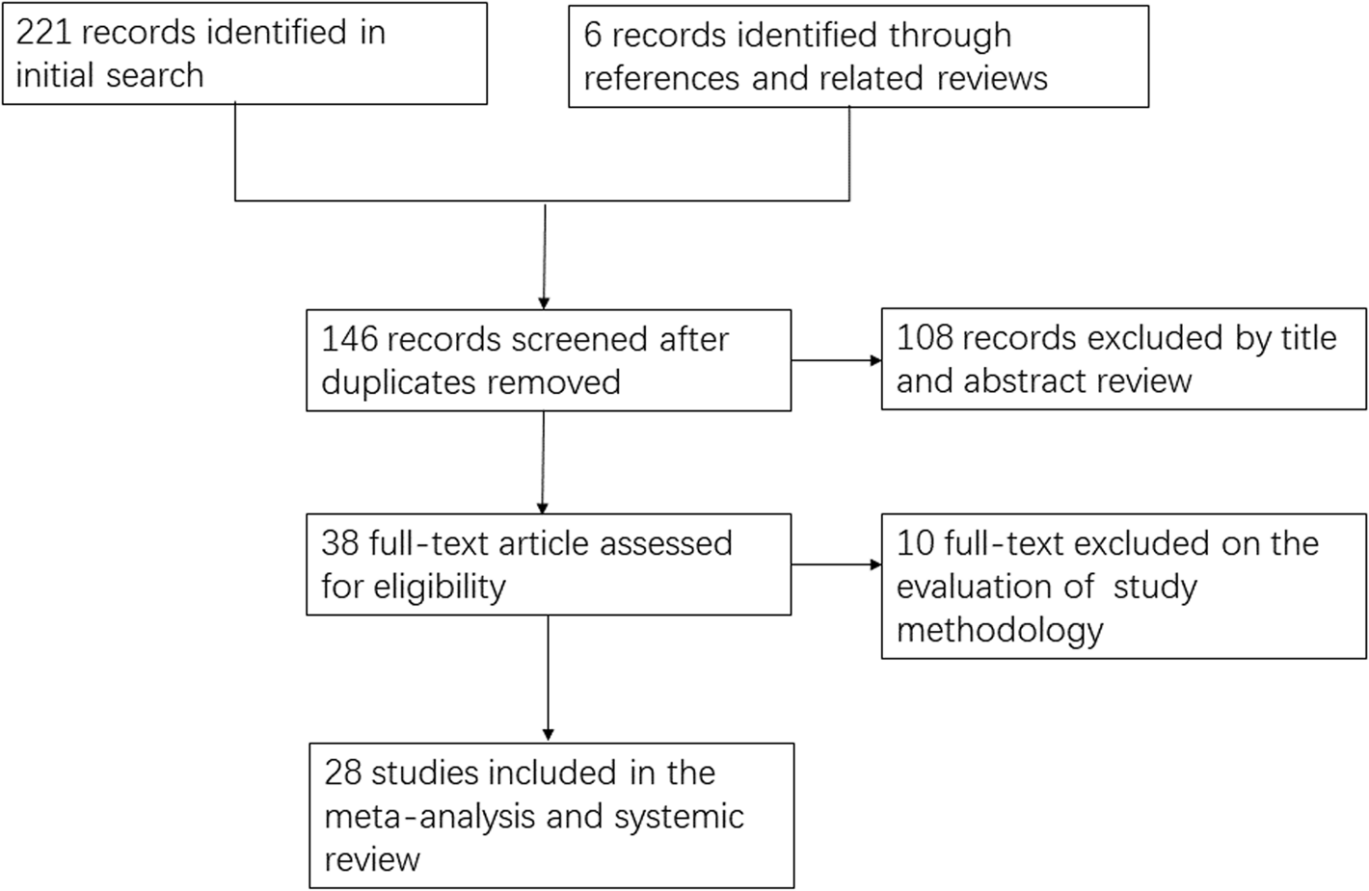
Flow diagram of the literature search strategy

Two hundred twenty-seven studies were identified from our initial search and 146 of them remained after duplicates were removed. One hundred eight of the remaining studies were excluded during title and abstract screening. Thirty-eight studies were identified for full-text assessment according to our inclusion and exclusion criteria and 10 of them were removed because of non-RCT, topical application of TXA or without placebo group. Finally, 28 studies [3, 4, 8, 10–12, 14–18, 23–39] were included in this meta-analysis. The study selection process is shown in Fig. 1.

### Description of included studies

The characteristics of included studies were shown in Table 1. The 28 included trials [3, 4, 8, 10–12, 14–18, 23–39] randomized 7446 patients (3712 to tranexamic acid and 3734 to placebo). Fourteen trials [4, 8, 11, 14–18, 25, 32, 36–39] randomized more than 99 patients. CABG was conducted in on-pump condition in 17 trails [12, 14, 16–18, 23–26, 28, 30–32, 34, 36, 38, 39], off-pump condition in 9 trails [3, 10, 11, 15, 27, 29, 33, 35, 37] and both condition in 2 trails [4, 8].

**Table 1:** Characteristics of included studies

Study ID	Country	No. C/T	Sex F/M	Type of GABG	AC discounted before surgery	Drug Dose and Treatment Regimens
Speekenbrink 1995 [23]	Netherlands	15/15	2/28	On-pump	2 to 4 days	TA 10 mg·kg^− 1^ in 20 min after induction of anesthesia and continued at a rate of 1 mg·kg^− 1^ up to a total dose of 1000 mg.
Brown 1997 [24]	United States	30/30	11/49	On-pump	NR	TA 15 mg·kg^− 1^ in 20 min after the induction and continued at a rate of 1 mg·kg^− 1^·hr.^− 1^ for 5 h
Landymore 1997 [25]	Canada	50/56	NR	On-pump	< 2 days	TA 10 mg·kg-1 before CBP and continued at a rate of mg·kg^− 1^·hr.^− 1^ until the termination of CBP
Hardy 1998 [26]	Canada	45/43	23/65	On-pump	NR	TA 10 g as a bolus over 20 min
Casati 2001 [27]	Italy	20/20	8/32	Off-pump	< 1 day	TA 1 g as a bonus before skin incision, followed by continuous infusion of 400 mg·hr.^− 1^ during surgery
Zabeeda 2002 [28]	Israel	25/25	12/38	On-pump	NR	TA 10 mg·kg^− 1^ in more than 15 min after induction of anesthesia and followed by a continuous infusion of 1 mg·kg^− 1^ per hour
Jares 2003 [29]	Czech Republic	22/25	12/35	Off-pump	5 days	TA 1 g as a bolus before skin incision, followed by continuous infusion of 200 mg·hr.^− 1^ during surgery
Pleym 2003 [30]	Norway	39/40	13/66	On-pump	1 day	TA 30 mg·kg^− 1^ as a bolus injection over 5 min immediately before the start of CPB.
Andreasen 2004 [31]	Denmark	23/21	7/37	On-pump	> 7 days	TA 1.5 g as a bolus, followed by a constant infusion of 200 mg·hr.^− 1^ until 1.5 g
Casati 2004 [4]	Italy	50/52	16/86	On-pump Off-pump	< 1 day	TA 1 g as a bonus before skin incision, followed by continuous infusion of 400 mg·hr.^− 1^ until completion of surgery with 500 mg added to priming in patients undergoing on-pump coronary artery bypass grafting
Karski 2005 [32]	Canada	165/147	37/275	On-pump	7 days	TA 100 mg·kg^− 1^ administered intravenously over 20 min after the induction of anesthesia
Vanek 2005 [33]	Czech Republic	30/32	14/38	Off-pump	< 1 day	TA 1 g before skin incision and a continuous infusion of 200 mg·hr.^− 1^ during the whole surgical procedure.
Santos 2006 [34]	Brasil	31/29	17/43	On-pump	NR	TA 10 mg·kg^− 1^ before the skin incision, followed by a continuous infusion of 1 mg·kg^− 1^·hr.^− 1^ for 5 h.
Wei 2006 [35]	China	40/36	16/60	Off-pump	5/−7 days	TA 0.75 g in 20 min at the beginning of surgery followed by continuous infusion of 0.25 g per hour throughout surgery.
Maddali 2007 [36]	Oman	111/111	70/152	On-pump	7 days	TA 10 mg·kg^− 1^ as a bolus prior to sternotomy, followed by an infusion (1 mg·kg^− 1^·hr.^− 1^) up to the time of starting of protamine.
Mehr-Aein 2007 [3]	Iran	33/33	2/27	Off-pump	7 days	TA 15 mg·kg^− 1^ before infusion of heparin and 15 mg·kg^− 1^ after protamine infusion
Taghaddomi 2009 [37]	Iran	50/50	28/72	Off-pump	NR	TA 1 g was given 20 min before skin incision and 400 mg·hr.^− 1^ during the entire surgical procedure.
Hashemi 2011 [38]	Iran	50/50	24/76	On-pump	NR	TA 1 g added to the pump prime solution and another 1 g was used intravenously after discontinuation of the pump
Ahn 2012 [10]	Korea	38/38	35/41	Off-pump	5 days	TA 1 g in 20 min before skin incision with subsequent continuous infusion at 200 mg·hr.^− 1^ during the operation
Chakravarthy 2012 [11]	India	50/50	22/78	Off-pump	7 days	TA 20 mg·kg^− 1^ over 30 min followed by infusion of 1 mg·kg^− 1^·hr.^− 1^ for 12 h
Greiff 2012 [12]	Norway	33/30	26/37	On-pump	1 day	TA 10 mg·kg-1 as a bolus injection before skin incision followed by an infusion of 1 mg·kg^− 1^·hr.^− 1^ until the end of surgery.
Nejad 2012 [14]	Iran	50/50	24/76	On-pump	NR	TA 1 g was added to the pump prime solution and another 1 g was used intravenously after the discontinuation of the pump
Wang 2012 [15]	China	115/116	36/195	Off-pump	5 days	TA 1 g as a bolus injection 20 min before the incision followed by an infusion of 400 mg·hr.^− 1^ until the completion of the surgery
Esfandiari 2013 [16]	Iran	75/75	30/120	On-pump	NR	TA 10 mg·kg^− 1^ added to the priming solution and a bolus dose of 1 mg·kg^− 1^ after weaning from CPB
Shi 2013 [17]	China	59/58	23/94	On-pump	< 7 days	TA 15 mg·kg^− 1^ before surgical incision and 15 mg·kg^− 1^ after protamine neutralization
Ghavidel 014 [39]	Iran	100/100	65/135	On-pump	3 days	TA 10 mg·kg^− 1^ via prime solution and the maintenance dose of 0.5–2 mg·kg^− 1^·h^− 1^ in proportion to serum creatinine.
Yanartas 2015 [18]	Turkey	63/69	50/82	On-pump	5 days	TA 10 mg·kg^− 1^ before the skin incision, followed by a continuous infusion of 1 mg·kg^− 1^·h^− 1^ for 5 h.
Myles 2017 [8]	Australia	2322/2311	773/3860	On-pump/ Off-pump	≥4 days	TA 100 mg·kg^− 1^ or 50 mg·kg^− 1^ was administered intravenously more than 30 min after the induction of anesthesia

### Risk of bias within studies

**Fig. 2:**
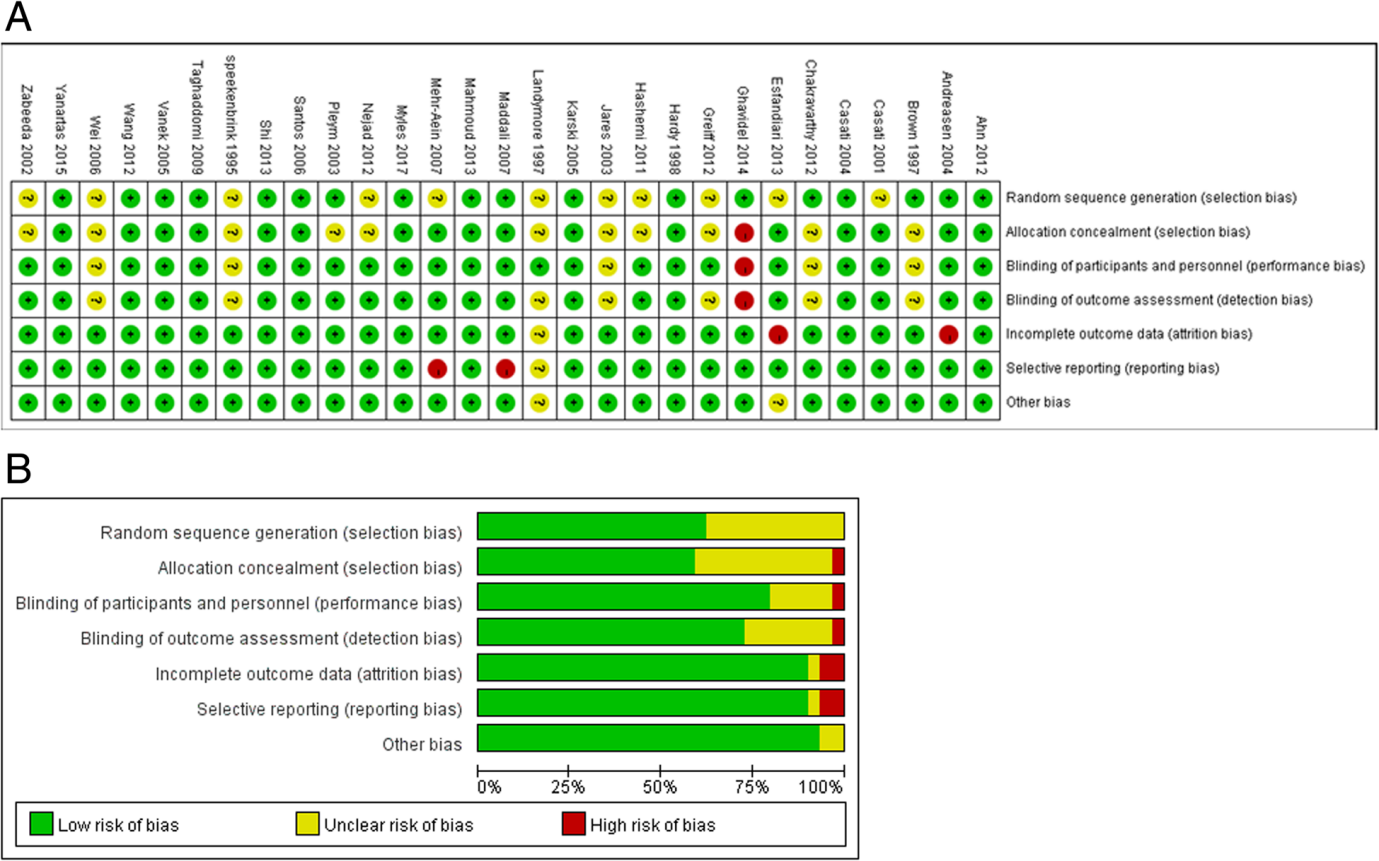
**a** risk-of-bias summary; **b** risk-of-bias graph for all the included randomized-controlled trials

The results of bias risk assessment were showed in Fig. 2a and b. Fourteen studies [3, 11, 12, 14, 16, 23–25, 27–30, 35, 38] did not provide a satisfactory description of their random processes. Blinding process was at high risk of bias in one study [39] and unclear risk of bias in 7 studies [11, 12, 23–25, 29, 35] due to unclear description. Three studies [16, 25, 31] had unclear or incomplete descriptions of their outcome data. Two studies [3, 36] were considered to be at high risk of selective reporting bias because the reported outcome indicators were inconsistent with the planed outcome indicators.

### Publication bias

Publication bias was evaluated by funnel plots in the following outcomes: postoperative cerebrovascular accident, reoperation for bleeding, mortality, myocardial infarction, acute renal insufficiency, the frequency of any allogeneic transfusions and 24-h postoperative chest tube drainage (Additional file 1: Figure S1, Additional file 2: Figure S2, Additional file 3: Figure S3, Additional file 4: Figure S4, Additional file 5: Figure S5 and Additional file 6: Figure S6 and Additonal file 7: Figure S7). All of the plots showed a symmetrical shape which suggested low risk of publication bias of the above outcomes.

### Quantitative data synthesis

#### Cerebrovascular accident

**Fig. 3:**
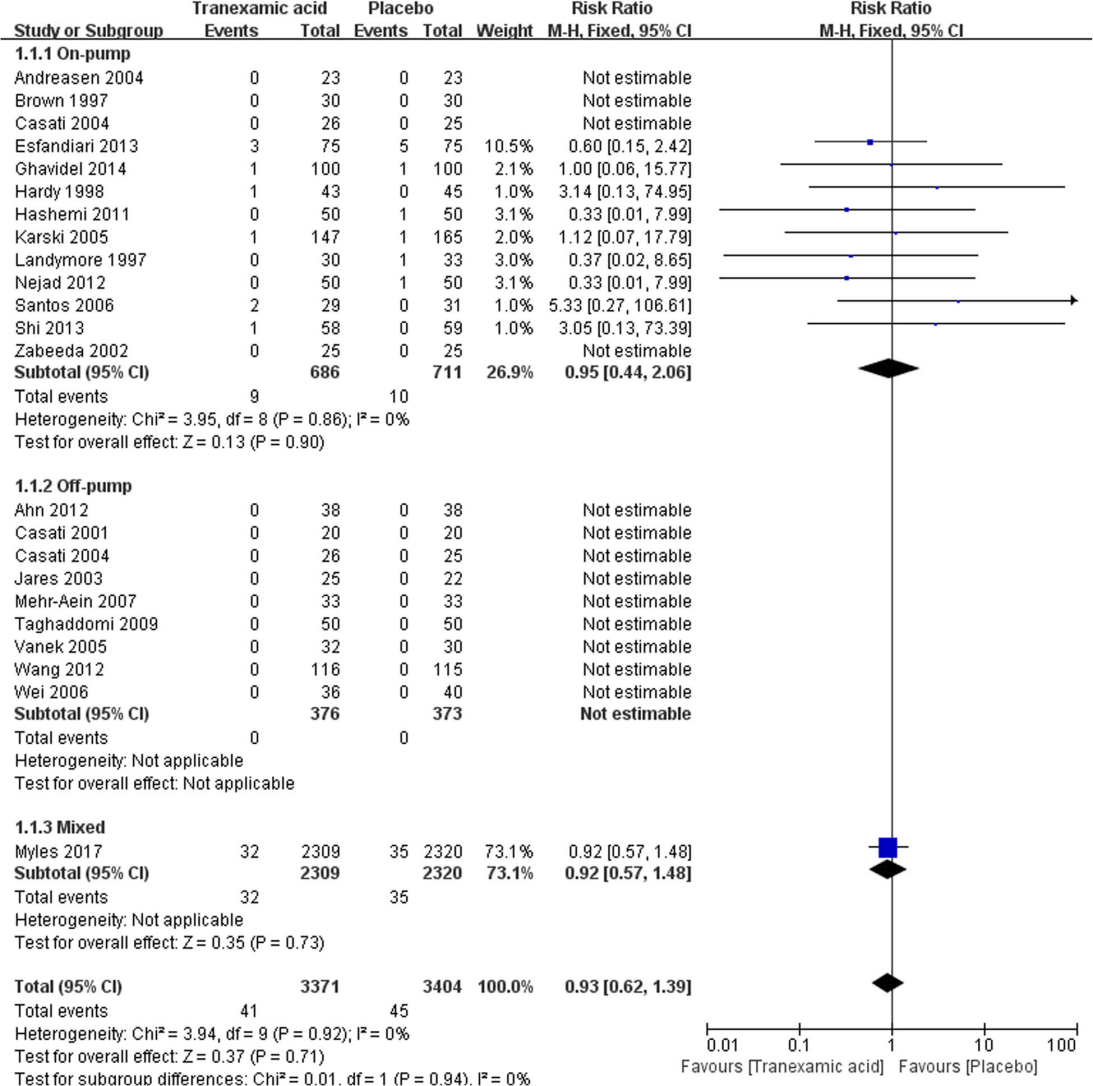
Forest plot of cerebrovascular accident

There were 22 trials that reported the incidence of postoperative cerebrovascular accident between TXA and placebo, with a total of 6775 participants. TXA did not increase the incidence of cerebrovascular accident overall from meta-analysis [41/3371 vs 45/3404, RR = 0.93(0.62–1.39), *P* for effect = 0.71, P for heterogeneity = 0.92, I^2^ = 0%] (Fig. 3).

Sub-analysis in on-pump CABG with 13 trials included showed no significant increase in the incidence of cerebrovascular accident in patients who received TXA treatment [9/686 vs 10/711, RR = 0.95(0.44–2.06), *P* for effect = 0.90, *P* for heterogeneity = 0.86, I^2^ = 0%]. In off-pump CABG, 8 trails with 749 participants were included and no cerebrovascular accident happened in those trials (Fig. 3).

Nine studies with a total of 5939 participants were included in the sensitive analysis of studies that randomized not less 100 participants. The conclusion that TXA would not increase cerebrovascular accident incidence was strengthened by the sensitivity analysis [RR = 0.87(0.57–1.33), *P* for effect = 0.53, *P* for heterogeneity = 0.95, I^2^ = 0%]. Sensitivity analysis that excluded the study with the largest number of participants furether strengthened the above conclusion [RR = 0.95(0.43–2.10), *P* for effect = 0.90, *P* for heterogeneity = 0.86] (Table 2).

**Table 2:** Sensitivity analysis of primary and secondary outcomes

Outcome	Sensitivity analyses	Studies (n)	TXA	Placebo	RR or MD	95% CI	*P* value for effect	P value for heterogeneity
Cerebrovascular accident	Studies randomized not less 100 patients	9	286/2999	318/3011	0.90	0.78–1.05	0.18	0.64
	Study with maximum sample size excluded	21	9/1062	10/1084	0.95	0.43–2.10	0.90	0.86
Reoperation for bleeding	Studies randomized not less 100 patients	8	29/2812	59/2821	0.49	0.32–0.77	< 0.01	0.58
	Study with maximum sample size excluded	15	17/815	30/814	0.59	0.34–1.04	0.07	0.72
Mortality	Studies randomized not less 100 patients	7	31/2870	36/2886	0.87	0.54–1.40	0.56	0.46
	Study with maximum sample size excluded	16	7/875	8/898	0.93	0.38–2.27	0.88	0.75
Myocardial infarction	Studies randomized not less 100 patients	11	286/2999	318/3011	0.90	0.78–1.05	0.18	0.64
	Study with maximum sample size excluded	22	23/1039	25/1045	0.94	0.55–1.61	0.81	0.8
Acute renal insufficiency	Studies randomized not less 100 patients	7	105/2758	102/2769	1.03	0.79–1.35	0.81	0.89
	Study with maximum sample size excluded	13	12/658	14/667	0.88	0.42–1.84	0.73	0.94
Transfusion of any blood products	Studies randomized not less 100 patients	7	954/2494	1400/2504	0.64	0.50–0.81	< 0.01	< 0.01
	Study with maximum sample size excluded	10	139/396	216/363	0.29	0.20–0.40	< 0.01	< 0.01
Postoperative chest tube drainage in the first 24 h	Studies randomized not less 100 patients	7	2824	2850	-208.3	−274.12,-142.48	< 0.01	< 0.01
	Study with maximum sample size excluded	17	802	814	−215.42	−259.48, −171.57	< 0.01	< 0.01

*TXA* tranexamic acid, *(n)* the number of cases, *RR* risk ratio, *MD* weighted mean difference, *CI* confidence interval

#### Seizures

**Fig. 4:**
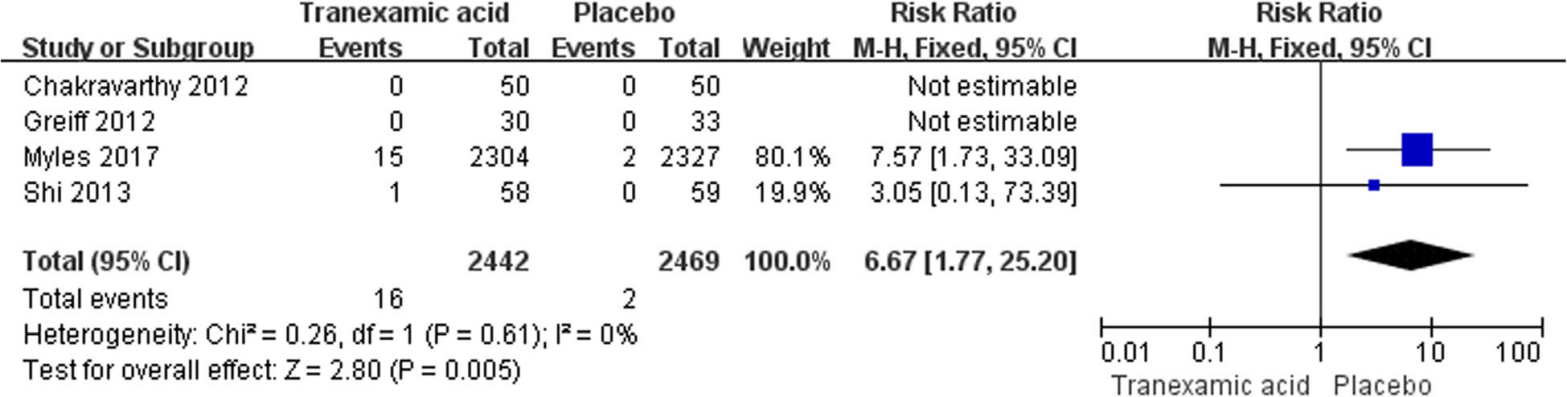
Forest plot of seizures

In total, 5 studies with 5043 participants reported the incidence of seizures after CABG. The summary RR for postoperative seizures with the use of TXA versus placebo was 5.99 (95% CI 1.77–20.24) which suggested that tranexamic acid would increase the incidence of seizures after CABG (Fig. 4).

#### Reoperation for bleeding

**Fig. 5:**
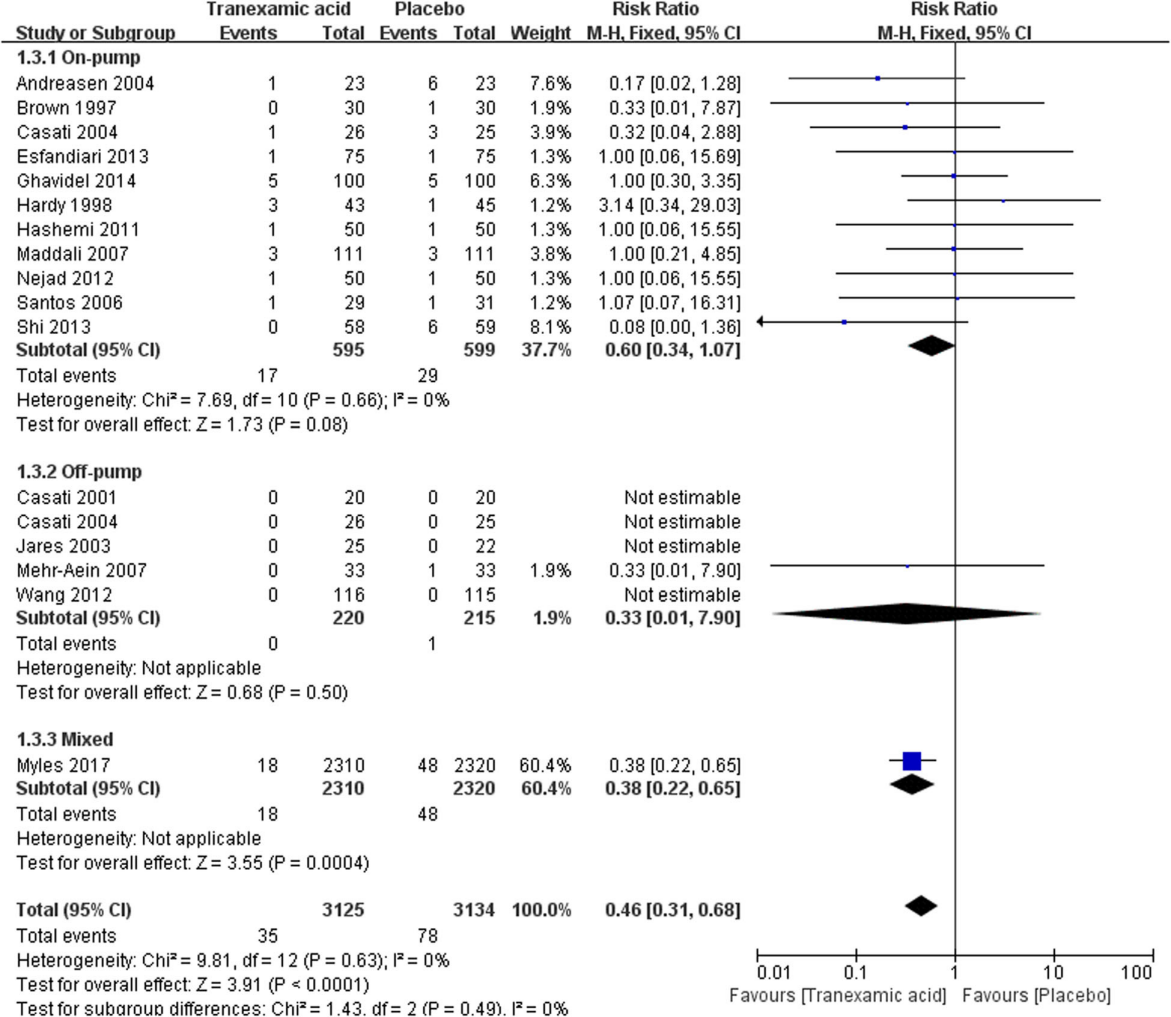
Forest plot of operation for bleeding

There were 16 trials that reported the incidence of postoperative reoperation for bleeding, with a total of 6259 participants. TXA decreased the incidence of reoperation for postoperative bleeding overall from meta-analysis [35/3125 vs 78/3134, RR = 0.46(0.31–0.68), *P* for effect< 0.01, *P* for heterogeneity = 0.63, I^2^ = 0%] (Fig. 5).

Ten studies with 1143 participants were included in on-pump CABG, the result of meta-analysis suggested no significant difference of reoperation for postoperative bleeding between TXA and placebo [16/569 vs 26/574, RR = 0.64 (0.35–1.15), *P* for effect = 0.14, *P* for heterogeneity = 0.62, I^2^ = 0%]. In off-pump subgroup, 4 studies with 384 participants were included and only one patient suffered reoperation in placebo group (Fig. 5).

Eight trials were included in sensitivity analysis of studies randomized not less than100 patients. The sensitivity analysis supported the result that TXA decreased incidence of reoperation for bleeding in CABG surgery when compared with placebo [29/2812 vs 59/2821, RR = 0.49 (0.32–0.77), *P* for effect< 0.01, *P* for heterogeneity = 0.58, I^2^ = 0%]. While sensitivity analysis that excluded the study with the largest number of participants did not supported the above conclusion [RR = 0.59 (0.34–1.04), *P* for effect = 0.07, *P* for heterogeneity = 0.72] (Table 2).

#### Mortality

**Fig. 6:**
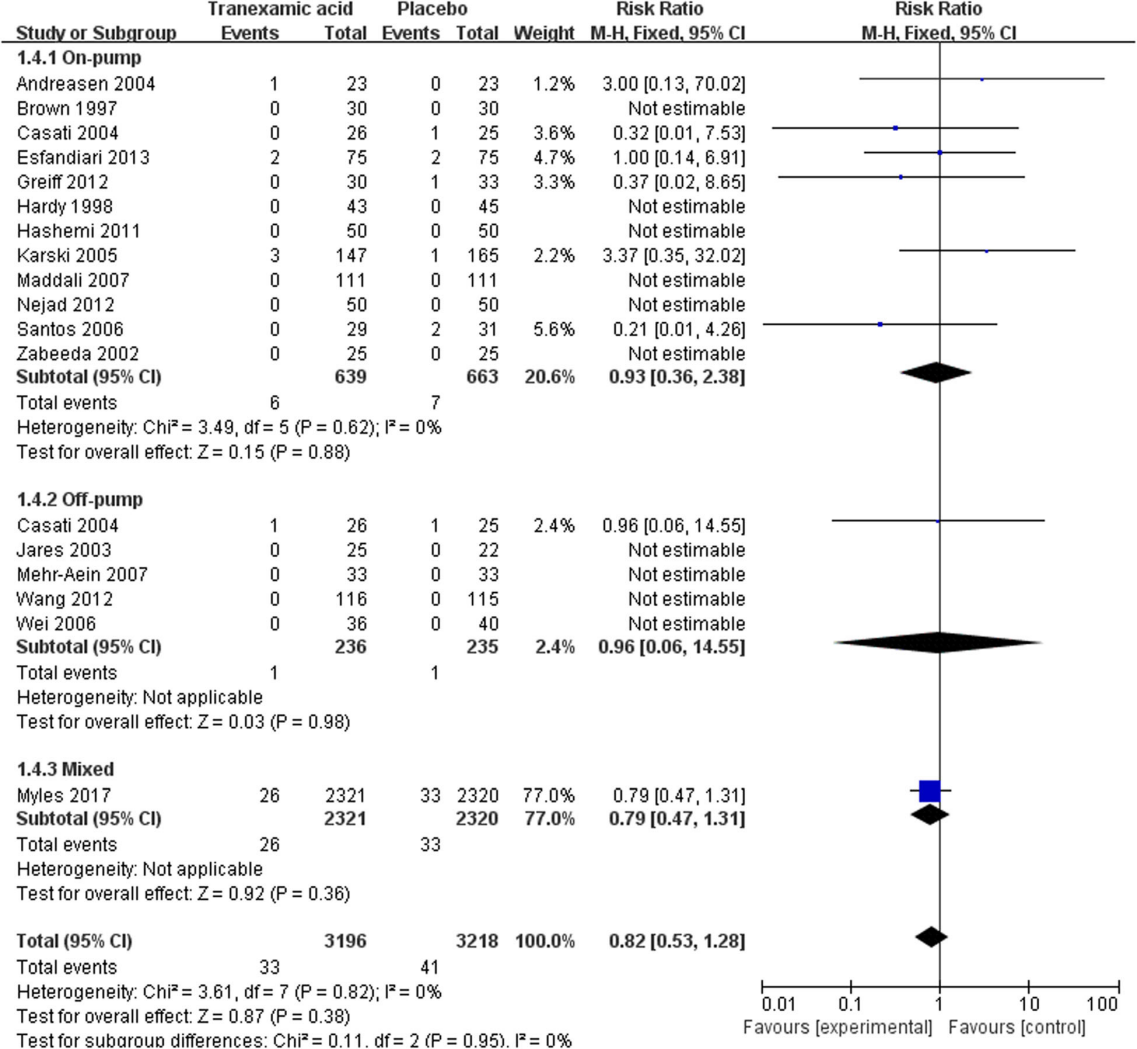
Forest plot of mortality

The overall analysis showed that TXA did not significantly decrease the mortality in patients receiving CABG when compared with placebo [33/3196 deaths in the TXA group vs 41/3218 deaths in the placebo group, RR = 0.82(0.53–1.28), *P* for effect = 0.38, *P* for heterogeneity = 0.82, I^2^ = 0%, with 18 trails included] (Fig. 6).

Sub-analysis in the settings of on-pump CABG also showed no statistically significant effect of TXA on mortality [6/639 vs 7/663, RR = 0.93 (0.36–2.38), *P* for effect = 0.88, *P* for heterogeneity = 0.62, I^2^ = 0%, with 12 trials included]. Sub-analysis in the settings of off-pump included 5 trials, but only one of them reported one patient died in each group (Fig. 6).

Sensitivity analysis of studies randomized more than 99 patients supported the results that TXA did not significantly decrease the mortality in CABG surgery compared with placebo [31/2870 vs 36/2886, RR = 0.87 (0.54–1.40), *P* for effect = 0.56, *P* for heterogeneity = 0.46, I^2^ = 0%, with 7 trials included]. The result of sensitivity analysis that excluded the study with maximum sample was consistent with the above analyses [7/875 vs 8/898, RR = 0.93 (0.38–2.27), *P* for effect = 0.88, *P* for heterogeneity = 0.75] (Table 2).

#### Myocardial infarction

**Fig. 7:**
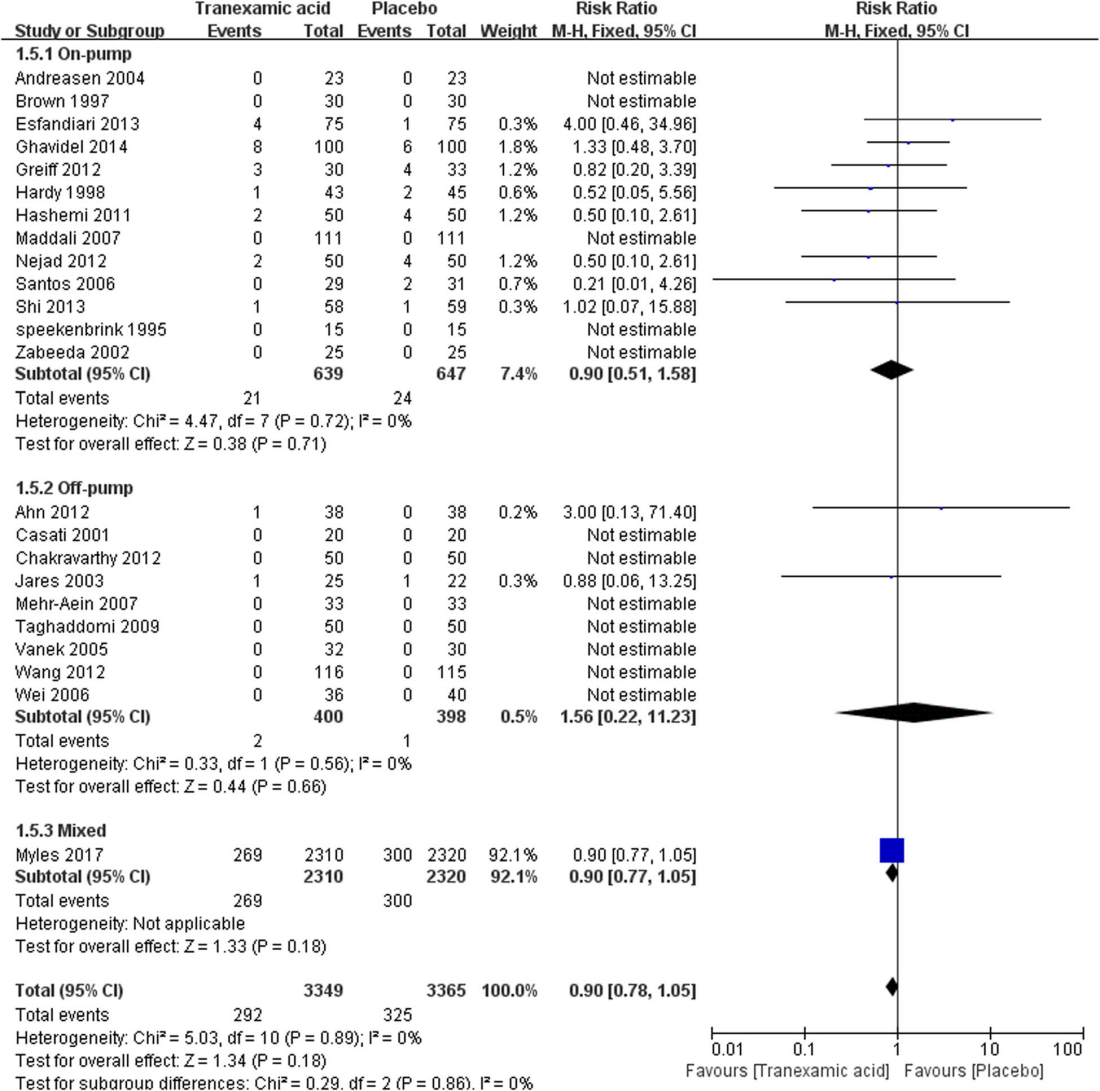
Forest plot of myocardial infarction

In total, 23 studies with 6714 participants reported the incidence of myocardial infarctions after CABG. The overall analysis showed no increased risk of postoperative myocardial infarction [292/3349 vs 325/3365, RR = 0.90 (0.78–1.05), *P* for effect = 0.18, *P* for heterogeneity = 0.89, I^2^ = 0%] (Fig. 7).

Thirteen studies with 1286 participants were included in the sub-analysis of on-pump CABG, the result of meta-analysis suggested no significant difference of myocardial infarction between TXA and placebo [21/639 vs 24/647, RR = 0.9 (0.51–1.58), *P* for effect = 0.71, *P* for heterogeneity = 0.72, I^2^ = 0%]. In off-pump subgroup, 9 studies with 798 participants were included, no significant difference of myocardial infarction between TXA and placebo was found neither [2/400 vs 1/398, RR = 1.56(0.22–11.23), *P* for effect = 0.66, *P* for heterogeneity = 0.56, I^2^ = 0%] (Fig. 7).

Seven trials were included in sensitivity analysis of studies randomized not less than100 patients. The sensitivity analysis supported the result that TXA did not increase myocardial infarction in CABG surgery when compared with placebo [286/2999 vs 318/3011, RR = 0.90 (0.78–1.05), *P* for effect = 0.18, *P* for heterogeneity = 0.64, I^2^ = 0%]. The result of sensitivity analysis that excluded the study with maximum sample was consistent with the above analyses [23/1039 vs 25/1045, RR = 0.94 (0.55–1.61), *P* for effect = 0.81, *P* for heterogeneity = 0.80] (Table 2).

#### Acute renal insufficiency

**Fig. 8:**
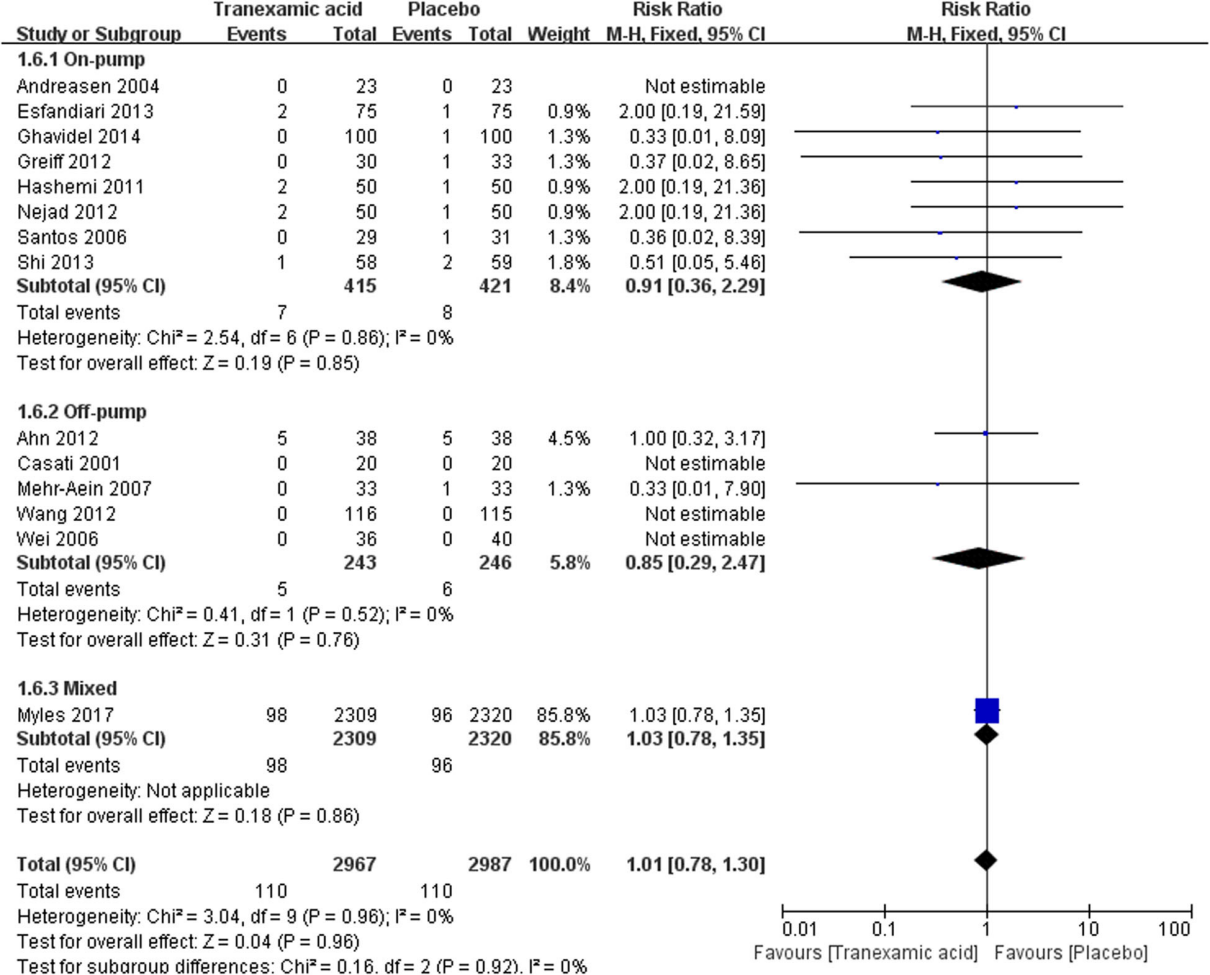
Forest plot of acute renal insufficiency

There are 14 studies that reported the incidence of acute renal insufficiency in this meta-analysis. The summary RR for acute renal with the use of TXA versus placebo was 1.01 (95% CI 0.77–1.32) which suggested that tranexamic acid would not increase the incidence of acute renal insufficiency (Fig. 8).

The summary RR of sub-analysis in on-pump CABG was 0.91 (95% CI 0.36–2.29) which suggested that TXA did not have adverse effect on postoperative renal function in patients undergoing on-pump CABG. A similar result was found in the sub-analysis in off-pump CABG [RR = 0.85 (0.29–2.47), *P* for effect = 0.76, *P* for heterogeneity = 0.52, I^2^ = 0%] (Fig. 8).

Sensitivity analysis in trials randomized not less than100 participants reinforced the overall analysis [RR = 1.03 (0.79–1.35), *P* for effect = 0.81, *P* for heterogeneity = 0.89, I^2^ = 0%, with 7 studies included]. The result of sensitivity analysis that excluded the study with maximum sample size was consistent with the above analyses [12/658 vs 14/667, RR = 0.88 (0.42–1.84), *P* for effect = 0.73, *P* for heterogeneity = 0.94] (Table 2).

#### Transfusion of any blood products

**Fig. 9:**
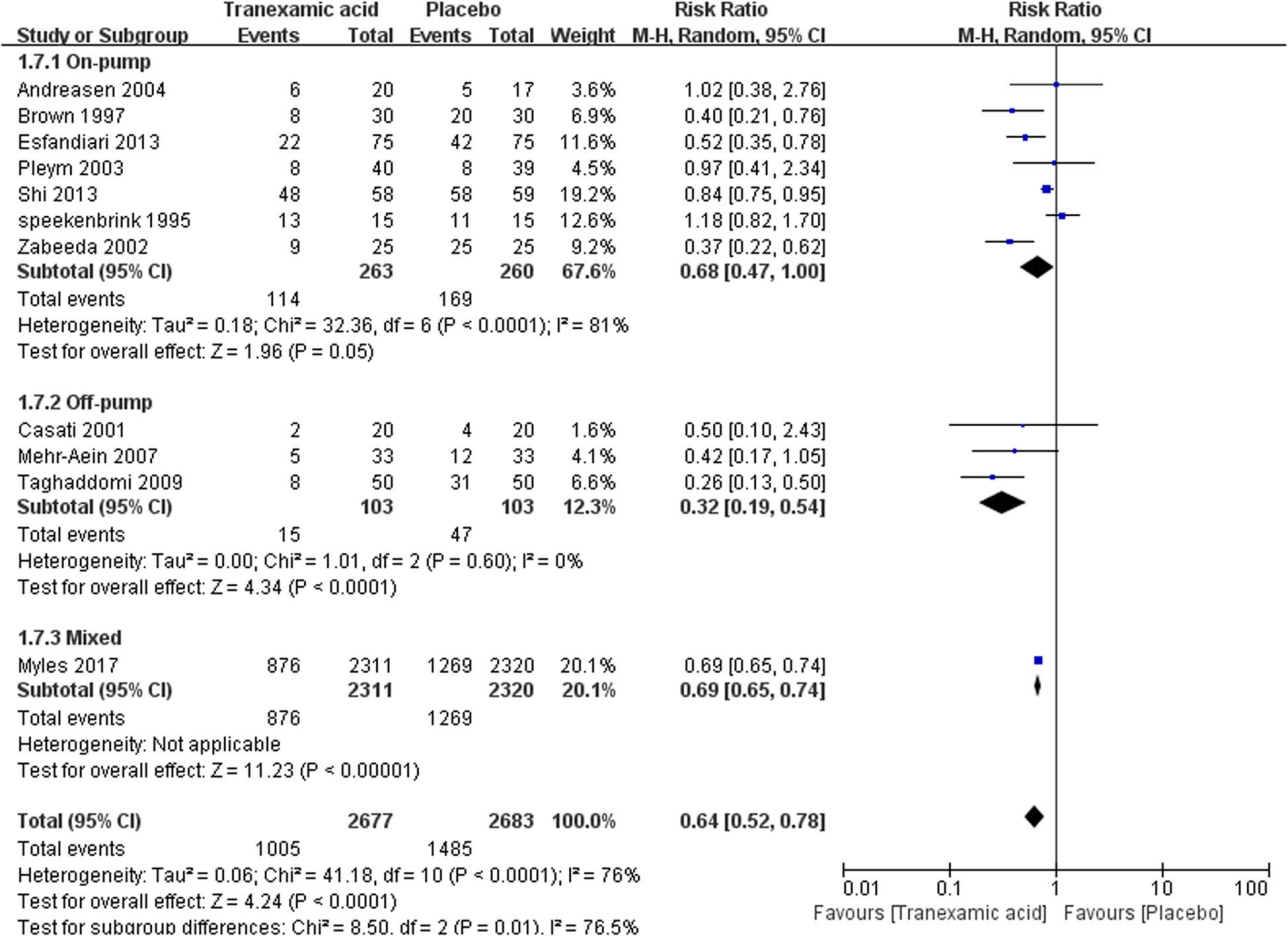
Forest plot of transfusion of any blood products

Eleven trails with a total of 5360 participants reported the postoperative transfusion rate of any blood product. Overall, TXA significantly reduced the transfusion of any blood products [RR = 0.64(0.52–0.78), *P* for effect< 0.01, *P* for heterogeneity< 0.01, I^2^ = 76%] (Fig. 9).

In the subgroup of patients undergoing on-pump CABG, TXA also reduced the transfusion of any blood products, however, this effect was not statistically significant [RR = 0.68(0.47–1.00), *P* for effect = 0.05, *P* for heterogeneity< 0.01, I^2^ = 81%]. On the other hand, sub-analysis in off-pump CABG, TXA significantly reduced the transfusion of any blood products [RR = 0.32(0.19–0.53), *P* for effect< 0.01, *P* for heterogeneity = 0.60, I^2^ = 0%] (Fig. 9).

In the sensitivity analysis that included all the studies that randomized more than 99 participants, TXA significantly decreased the transfusion of any blood products [RR = 0.64(0.50–0.81), *P* for effect< 0.01, *P* for heterogeneity< 0.01, I^2^ = 86%]. The result of sensitivity analysis that excluded the study with maximum sample size further enhanced the above analyses [139/396 vs 216/363, RR = 0.29 (0.20–0.40), *P* for effect < 0.01, *P* for heterogeneity < 0.01] (Table 2).

#### Postoperative chest tube drainage in the first 24 h

**Fig. 10:**
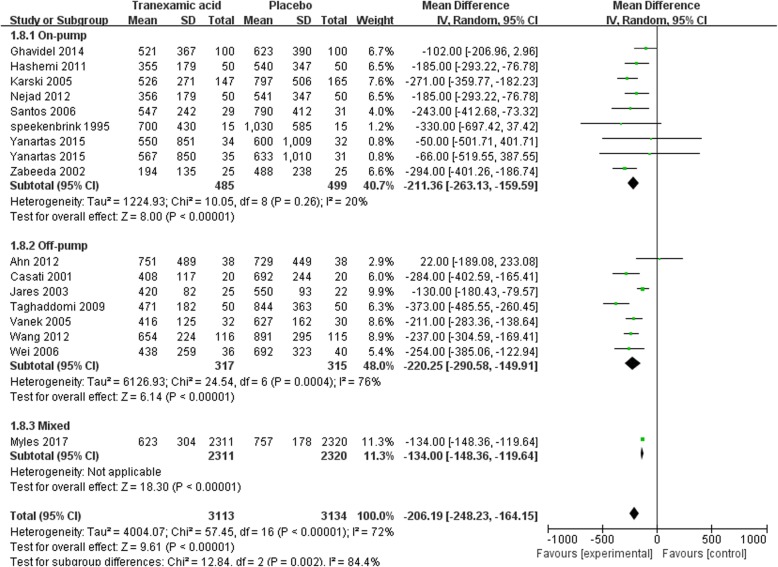
Forest plot of chest tube drainage in the first 24 h

In total, 16 studies with 6247 participants were included in the meta-analysis of postoperative chest tube drainage in the first 24 h. One of them [18] divided participants into two groups according to the difference in fluid use and reported the drainage of patients receiving TXA and placebo in both groups separately. We treated these two sets of data as two separate studies in the meta-analysis. Overall, the chest tube drainage was significantly decreased by TXA when compared with placebo [MD = -206.19, 95% CI (− 248.23, − 164.15), *P* for effect< 0.01, *P* for heterogeneity< 0.01, I^2^ = 72%] (Fig. 10).

Sub-analysis in the settings of on-pump CABG with 8 trials included showed no significant decrease of chest tube drainage in the first 24 h in patients who received TXA treatment [MD = -211.36, 95% CI (− 263.13, − 159.59), *P* for effect< 0.01, *P* for heterogeneity = 0.26, I^2^ = 20%]. A similar result was found in the sub-analysis in off-pump CABG [MD = -220.25, 95% CI (− 290.58, − 149.91), *P* for effect< 0.01, *P* for heterogeneity = 0.26, I^2^ = 76%] (Fig. 10).

Seven studies with a total of 5674 participants were included in the sensitive analysis. The conclusion that TXA would decrease chest tube drainage in the first 24 h was strengthened by the sensitivity analysis [MD = − 208.30, 95% CI (− 274.12, − 142.48), *P* for effect< 0.01, *P* for heterogeneity< 0.01, I^2^ = 83%]. The sensitivity analysis that excluded the study with maximum sample size also supported the above conclusion [MD = -215.42, 95% CI (− 259.48, − 171.57), *P* for effect < 0.01, *P* for heterogeneity< 0.01, I^2^ = 83%] (Table 2).

#### Quality of the evidence

The GRADE approach was adopted to evaluate the quality of each outcome and “Summary of findings” tables were presented (Table 3). In general, the overall quality of evidence in the meta-analyses of postoperative seizures and reoperation for bleeding was high. However, the overall quality of evidence in the meta-analyses of postoperative transfusion of any blood products and chest tube drainage in the first 24 h was very low due to the problems of inconsistency and the risk of bias. The overall quality of evidence of other outcomes was moderate due to the risk of bias.

**Table 3:** GRADE summary of findings table

Outcomes	Illustrative comparative risks^a^ (95% CI)		Relative effect (95% CI)	No of Participants (studies)	Quality of the evidence (GRADE)	Comments
	Assumed risk Control	Corresponding risk Tranexamic acid versus placebo				
Cerebrovascular accident	Study population		RR 0.93 (0.62 to 1.39)	6775 (22 studies)	⊕ ⊕ ⊕⊝ moderate^b^	
	13 per 1000	12 per 1000 (8 to 18)				
	Moderate					
	0 per 1000	0 per 1000 (0 to 0)				
Seizure	Study population		RR 6.67 (1.77 to 25.20)	4911 (4 studies)	⊕ ⊕ ⊕ ⊕ high^c,d^	
	1 per 1000	5 per 1000 (1 to 20)				
	Moderate					
	0 per 1000	0 per 1000 (0 to 0)				
Reoperation for bleeding	Study population		RR 0.46 (0.31 to 0.68)	6259 (16 studies)	⊕ ⊕ ⊕ ⊕ high^e,f^	
	25 per 1000	11 per 1000 (8 to 17)				
	Moderate					
	22 per 1000	10 per 1000 (7 to 15)				
Mortality	Study population		RR 0.82 (0.53 to 1.28)	6414 (17 studies)	⊕ ⊕ ⊕⊝ moderate^b,g^	
	13 per 1000	10 per 1000 (7 to 16)				
	Moderate					
	0 per 1000	0 per 1000 (0 to 0)				
Myocardial infarction	Study population		RR 0.9 (0.78 to 1.05)	6714 (23 studies)	⊕ ⊕ ⊕⊝ moderate^e^	
	97 per 1000	87 per 1000 (75 to 101)				
	Moderate					
	0 per 1000	0 per 1000 (0 to 0)				
Acute renal insufficiency	Study population		RR 1.01 (0.78 to 1.3)	5954 (14 studies)	⊕ ⊕ ⊕⊝ moderate^b^	
	37 per 1000	37 per 1000 (29 to 48)				
	Moderate					
	20 per 1000	20 per 1000 (16 to 26)				
Transfusion of any blood products	Study population		RR 0.64 (0.52 to 0.78)	5360 (11 studies)	⊕⊝⊝⊝ very low^b,h^	
	553 per 1000	354 per 1000 (288 to 432)				
	Moderate					
	560 per 1000	358 per 1000 (291 to 437)				
Postoperative chest tube drainage in the first 24 h		The mean postoperative chest tube drainage in the first 24 h in the intervention groups was 206.19 lower (248.23 to 164.15 lower)		6247 (16 studies)	⊕⊝⊝⊝ very low^h,i^	

GRADE Working Group grades of evidence

High quality: Further research is very unlikely to change our confidence in the estimate of effect

Moderate quality: Further research is likely to have an important impact on our confidence in the estimate of effect and may change the estimate

Low quality: Further research is very likely to have an important impact on our confidence in the estimate of effect and is likely to change the estimate

Very low quality: We are very uncertain about the estimate

*CI* Confidence interval, *RR* Risk ratio, *OR* Odds ratio

^a^The basis for the assumed risk (e.g. the median control group risk across studies) is provided in footnotes. The corresponding risk (and its 95% confidence interval) is based on the assumed risk in the comparison group and the relative effect of the intervention (and its 95% CI)

^b^4 studies with a high risk of bias were included

^c^few studies reported this result

^d^RR > 5

^e^5 studies with a high risk of bias were included

^f^RR < 0.5

^g^No explanation was provided

^h^I2 > 75%

^i^2 studies with a high risk of bias were included

## Discussion

In this meta-analysis, we found that the intravenous use of TXA was associated with lower risk of reoperation for postoperative bleeding, blood loss and blood transfusion than the placebo group. Moreover, we also found that intravenous use of TXA did not increase the risk of postoperative cerebrovascular accident, mortality or other thrombotic complication among patients undergoing CABG when compared with placebo treatment. However, it may increase the incidence of postoperative seizures. The results of most subgroup analyses of the primary results in CABG conducted under on-pump or off-pump condition were consistent with that of overall analyses. However, meta-analysis could not be performed in the sub-analyses of postoperative reoperation for bleeding, mortality and cerebrovascular accident in off-pump CABG due to the small number of incidence. No significant decrease in postoperative reoperation for bleeding and transfusion of any blood products were found in on-pump group. Most of the sensitivity analyses in trails that recruited more than 99 participants or in trails that excluded the study with the largest number of participants further strengthened the conclusion of overall analyses.

The release of plasmin during cardiac surgery activates fibrinolysis and may contribute to platelet dysfunction [40]. In addition to inhibiting the transformation of plasminogen into plasmin by reversibly binding lysine binding site on plasmin, TXA can also reduce bleeding by preventing platelet activation induced by fibrinolytic enzyme [41]. A previous meta-analysis suggested that TXA was effective in reducing blood loss and the need for blood transfusion in cardiac surgery [42]. However, the incidence of reoperation for bleeding was not significantly decrease by TXA [42]. In our current analysis, we found that TXA overall reduced the transfusion of any blood products and 24-h postoperative chest tube drainage in CABG surgery which was consistent with the previous study. Moreover, the sub-analyses in the different conditions under which GABG was conducted further strengthened the above results. However, these analyses have significant heterogeneity which may due to the difference in indications of blood transfusion, drug dose and treatment regimens among different studies.

Different from the previous study, our current mete-analysis suggested that TXA significantly decrease the incidence of reoperation for bleeding in CABG surgery with low heterogeneity. In addition, the sensitivity analyses in studies randomized more than 99 participants further strengthened the conclusion that TXA reduced the incidence of reoperation for bleeding, transfusion of any blood products and 24-h blood loss suggesting that the small sample size studies included in the meta-analysis did not affect the overall effectiveness. However, the sensitivity analysis that excluded the study [8] with maximum sample size did not suggest that TXA would significantly decrease the incidence of reoperation for bleeding. This result suggested that the study with the largest number of participants largely determines the overall effect of meta-analysis. While considering the low risk of bias assessment in that study, we can still believe that TXA overall decrease the incidence of reoperation for bleeding. In the sub-analysis of on-pump GABG, TXA tended to reduce the incidence of reoperation for bleeding. However, the effect was not statistically significant. The exclusion of the study with the largest number of participants due to mixed surgical types in the sub-analysis may explain this difference.

Although lots of studies have suggested that blood transfusion and reoperation for bleeding is associated with poor outcomes after cardiac surgery, we did not find that TXA would reduce the risk of cerebrovascular accident, myocardial infarction, acute renal insufficiency or mortality despite its effectiveness in reducing transfusion and reoperation for bleeding. A previous meta-analysis had reported that TXA reduced blood transfusion in off-pump CABG and did not increased the incidence of postoperative adverse events [9]. However, the sample size in that study was not sufficient to detect the rare but clinically significant adverse events. In the current meta-analysis, enough population were included in the above analyses to detect clinically significant difference. Moreover, the above conclusion were strengthened by sensitivity analyses in trails enrolling more than 99 patients or sensitivity analyses excluded the study with largest sample size. In addition, there was no heterogeneity in above analyses from the results of heterogeneity tests and the risk of publication bias in these meta-analyses was quite low revealed by funnel plots. These unexpected results may be explained by the potential prothrombotic effects of TXA. It is well known that 5 to 15% of all grafts may be blocked in the early postoperative period even without the use of antifibrinolytic agents, which may led to recurrence of myocardial ischemia, infarction, or even death [43, 44]. Perioperative inhibition of fibrinolysis may increase the rate of early graft occlusion rate [45]. The phenomenon that TXA reduced transfusion, blood loss and incidence of reoperation without decreasing postoperative morality or adverse events may be a balance of its blood conservation effect and potential prothrombotic effect.

A previous meta-analysis suggested that the risk of seizure increased in patients with TXA exposure [46]. In the current meta-analysis we found that TXA increased the incidence of postoperative seizures in CABG surgery. Several studies have suggested that the convulsant property of TXA is likely mediated by disinhibition of gama-aminobutyric acid type A (GABAA) receptors and glycine receptor, which are two major mediators of inhibition in the CNS [47, 48]. Moreover, TXA did not interfere with N-methyl-Daspartate receptor and impact glutamatergic synaptic transmission [48, 49]. In addition, some studies have shown that TXA reduces cerebral blood flow and increases the risk of cerebral infarction which could contribute to the postoperative seizures. However, the meta-analysis of postoperative cerebrovascular accident in current study did not supported the hypothesis that TXA increase incidence of seizures by increasing the incidence of cerebral infarction. Moreover, a growing number of studies have suggested the seizures associated with TXA to be dose related [6, 50, 51]. Therefore, studies that investigate the optimize dose and regime for administration of TXA are needed in the future. Moreover, a growing number of studies that investigate the efficacy and safety of topical use of tranexamic acid have been conducted in recent years due to the promise of reducing postoperative bleeding and seizures [52, 53]. A recent meta-analysis showed that the topical application of TXA effectively reduces both transfusion risk and blood loss compared to placebo and no major differences were found between topical and intravenous tranexamic acid with respect to safety and efficacy [54]. However, both surgical and non-surgical trials were included in that study. While in our study, we focused on the safety and efficiency of intravenous administration of tranexamic acid in coronary artery bypass grafting (CABG).

There are some limitations in this meta-analysis. Firstly, heterogeneity due to clinical and methodological diversity was inevitable which may affect the reliability of the analysis results especially in meta-analyses of transfusion and blood loss. Secondly, some data were presented as median and interquartile range which cannot be used in performing meta-analysis. We estimated the mean and standard deviation from those data to perform meta-analysis which may compromise the reliability of analysis results. Thirdly, the postoperative incidence of adverse event was suggested to may be dose-dependent [6], while we failed to performed sub-analysis in different dose setting due to the various dosage and regimens of TXA administration in current meta-analysis. Fourthly, a multicenter study that randomized 2311 participants occupied the main part of most analyses which may lead to bias. Despite the above limitations, the current study is still the most comprehensive analysis on the efficacy and safety of TXA in CABG surgery with sufficient sample size.

## Conclusion

The current study systematically reviewed the existing evidence on the efficacy and safety profile of the intravenous administration of TXA in CABG surgery and showed that TXA would significantly reduce postoperative transfusion of any blood products, 24-h postoperative chest tube drainage and reoperation for bleeding. In addition, our results identified for the first time that intravenous administration of TXA in CABG surgery did not increase the risk of prothrombotic complication with sufficient sample size. However, it may increase the risk of postoperative seizures. Overall, intravenous administration of TXA in CABG surgery is effective and safe in reducing blood loss and transfusion according to the existing evidence and further studies are needed to identify the optimal dose and regime for intravenous use of TXA to achieve the best benefit with lowest risk.

## Additional files

Additional file 1: **Figure S1.** Funnel plot of cerebrovascular accident (PNG 8 kb)

Additional file 2: **Figure S2.** Funnel plot of reoperation for bleeding (PNG 8 kb)

Additional file 3: **Figure S3.** Funnel plot of mortality (PNG 8 kb)

Additional file 4: **Figure S4.** Funnel plot of myocardial infarction (PNG 8 kb)

Additional file 5: **Figure S5.** Funnel plot of acute renal insufficiency (PNG 8 kb)

Additional file 6: **Figure S6.** Funnel plot of transfusion of any blood products (PNG 7 kb)

Additional file 7: **Figure S7.** Funnel plot of chest tube drainage in the first 24 h (PNG 5 kb)

## Data Availability

All data generated or analysed during this study are included in this published article and its supplementary information files.

## Abbreviations

CABG: Coronary artery bypass grafting
CNS: Central nervous system
GABAA: Gama-aminobutyric acid type A
TXA: Tranexamic acid
