# Supplementary figures and images for: The safety and efficiency of intravenous administration of tranexamic acid in coronary artery bypass grafting (CABG): a meta-analysis of 28 randomized controlled trials
Source: BMC Anesthesiol. 2019 Jun 14;19:104. doi: 10.1186/s12871-019-0761-3 (PMC6567423; doi:10.1186/s12871-019-0761-3)

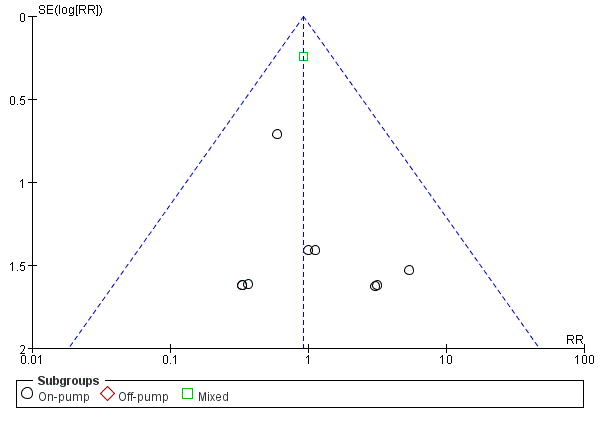

Supplement: Supplementary file 1 — Figure S1. Funnel plot of cerebrovascular accident (PNG 8 kb) [file 12871_2019_761_MOESM1_ESM.png]

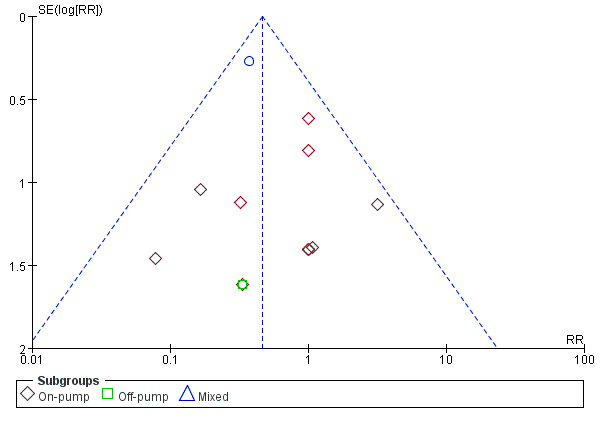

Supplement: Supplementary file 2 — Figure S2. Funnel plot of reoperation for bleeding (PNG 8 kb) [file 12871_2019_761_MOESM2_ESM.png]

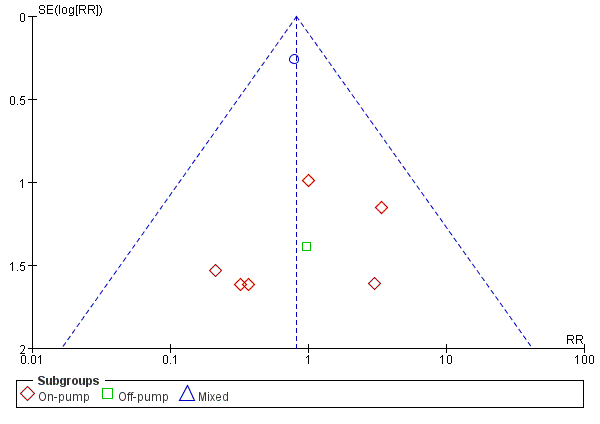

Supplement: Supplementary file 3 — Figure S3. Funnel plot of mortality (PNG 8 kb) [file 12871_2019_761_MOESM3_ESM.png]

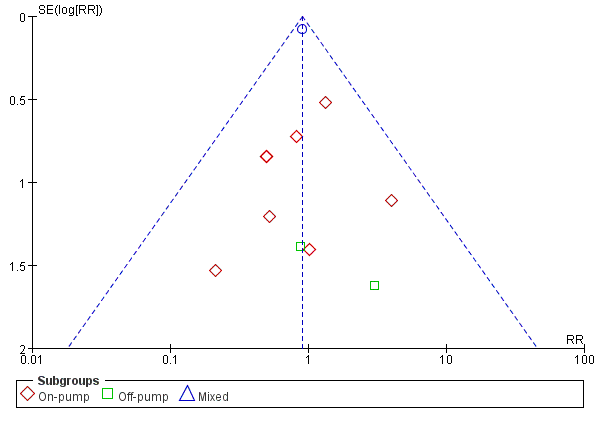

Supplement: Supplementary file 4 — Figure S4. Funnel plot of myocardial infarction (PNG 8 kb) [file 12871_2019_761_MOESM4_ESM.png]

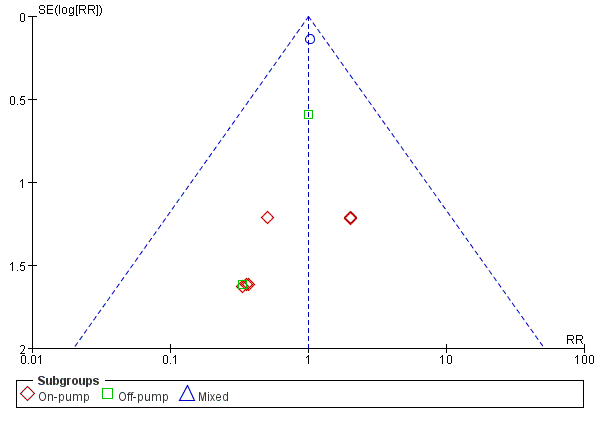

Supplement: Supplementary file 5 — Figure S5. Funnel plot of acute renal insufficiency (PNG 8 kb) [file 12871_2019_761_MOESM5_ESM.png]

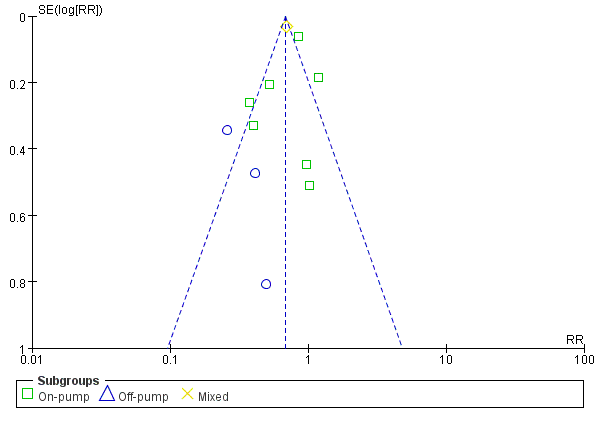

Supplement: Supplementary file 6 — Figure S6. Funnel plot of transfusion of any blood products (PNG 7 kb) [file 12871_2019_761_MOESM6_ESM.png]

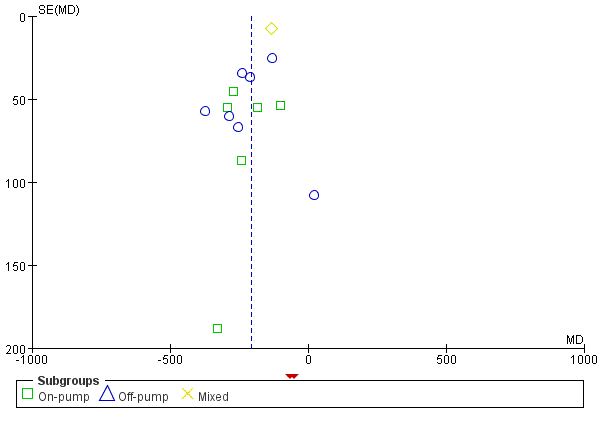

Supplement: Supplementary file 7 — Figure S7. Funnel plot of chest tube drainage in the first 24 h (PNG 5 kb) [file 12871_2019_761_MOESM7_ESM.png]
